# Supplementary material for: Cationic Cellulose Nanocrystals-Based Nanocomposite Hydrogels: Achieving 3D Printable Capacitive Sensors with High Transparency and Mechanical Strength
Source: Polymers (Basel). 2021 Feb 25;13(5):688. doi: 10.3390/polym13050688 (PMC7956583; doi:10.3390/polym13050688)
Supplement: Supplementary file 1 [file polymers-13-00688-s001.pdf]

polymers

MDPI

Article

Cationic Cellulose Nanocrystals-Based Nanocomposite Hydrogels: Achieving 3D Printable Capacitive Sensors with High Transparency and Mechanical Strength

Po-Cheng Lai and Sheng-Sheng Yu *

Department of Chemical Engineering, National Cheng Kung University, No. 1 University Road, Tainan 70101, Taiwan; N36094136@gs.ncku.edu.tw
* Correspondence: ssyu@mail.ncku.edu.tw; Tel.: +886-6-2757575 ext. 62628

Citation: Lai, P.-C.; Yu, S.-S. Cationic Cellulose Nanocrystals-based Nanocomposite Hydrogels: Achieving 3D Printable Capacitive Sensors with High Transparency and Mechanical Strength. *Polymers* **2021**, *13*, 688. https://doi.org/10.3390/polym13050688

Academic Editor: Constantinos Tsitsilianis

Received: 31 January 2021
Accepted: 23 February 2021
Published: 25 February 2021

Publisher's Note: MDPI stays neutral with regard to jurisdictional claims in published maps and institutional affiliations.

Copyright: © 2021 by the authors. Licensee MDPI, Basel, Switzerland. This article is an open access article distributed under the terms and conditions of the Creative Commons Attribution (CC BY) license (http://creativecommons.org/licenses/by/4.0/).

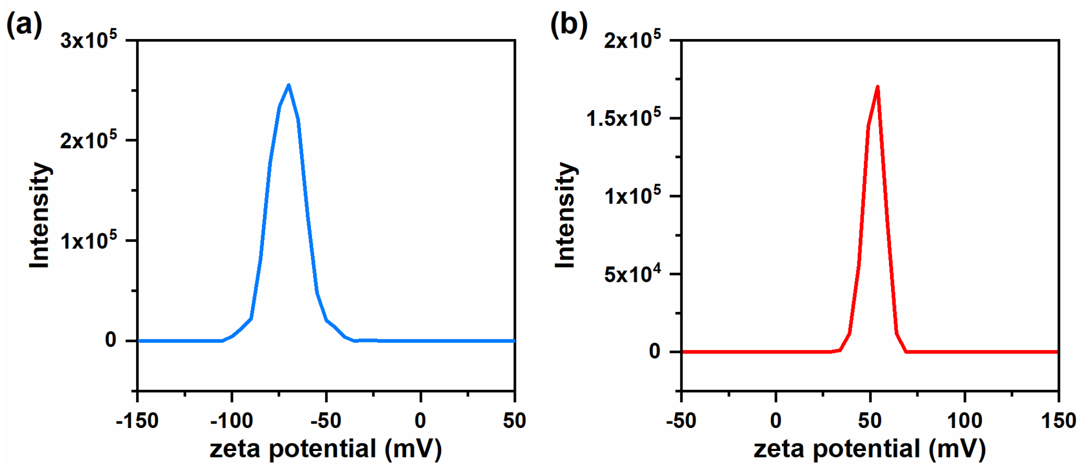

Figure S1. Zeta potential profiles of (**a**) CNCs. (**b**) CCNCs.

Table S1. Elemental analysis of CNCs and CCNCs.

Sample	Elemental composition (%)				
	N	C	H	S	O
CNCs	0	40.38	6.69	0.51	52.42
CCNCs	0.94	40.60	7.27	0.55	50.64

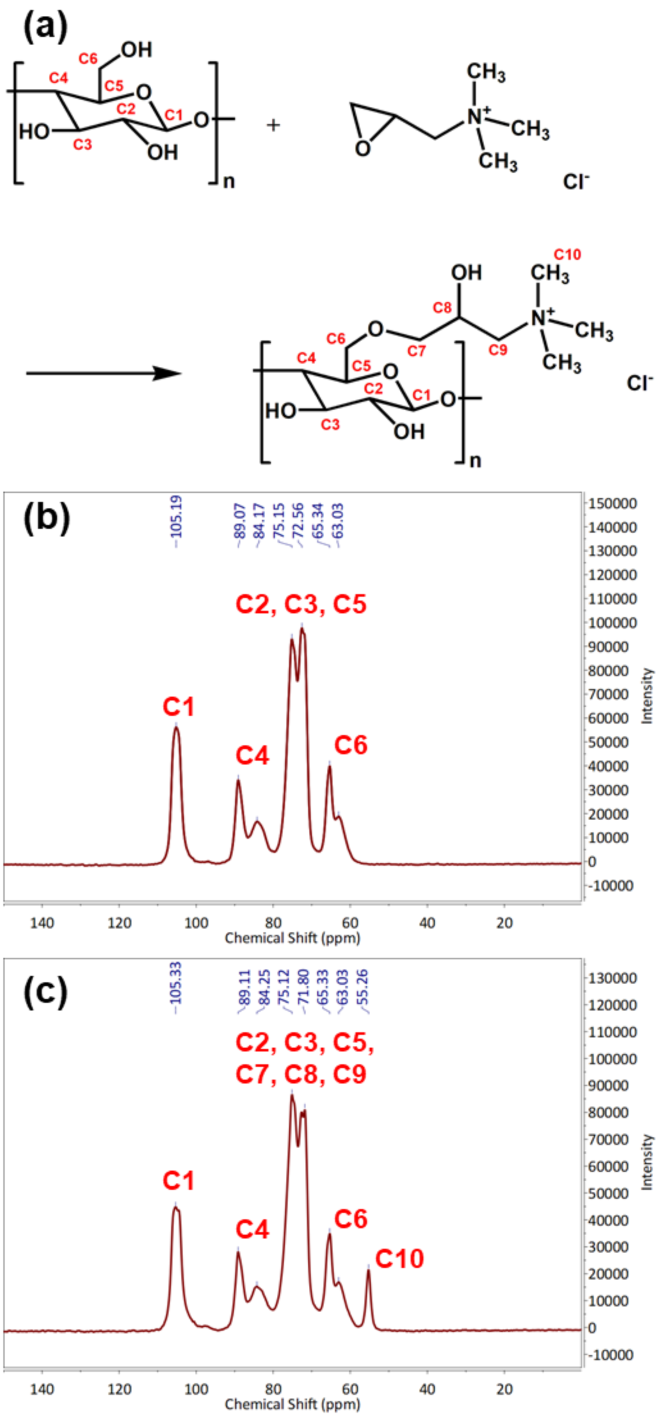

Figure S2. (**a**) Reaction between CNCs and EPTMAC. (**b**) Solid-state ^{13}C NMR spectrum of CNCs. (**c**) Solid-state ^{13}C NMR spectrum of CCNCs. Similar to the spectra reported in literature[1,2], we found the following peaks for CNCs: 105.2 ppm (C1 crystalline), 89.1 ppm (C4 crystalline), 84.2 ppm (C4 amorphous), 75.1/71.8 ppm (C2/C3/C5), 65.3 ppm (C6 crystalline), 63.0 ppm (C6 amorphous). For CCNCs, a new peak for the methyl groups of the grafted EPTMAC was found at 55.3 ppm (C10).

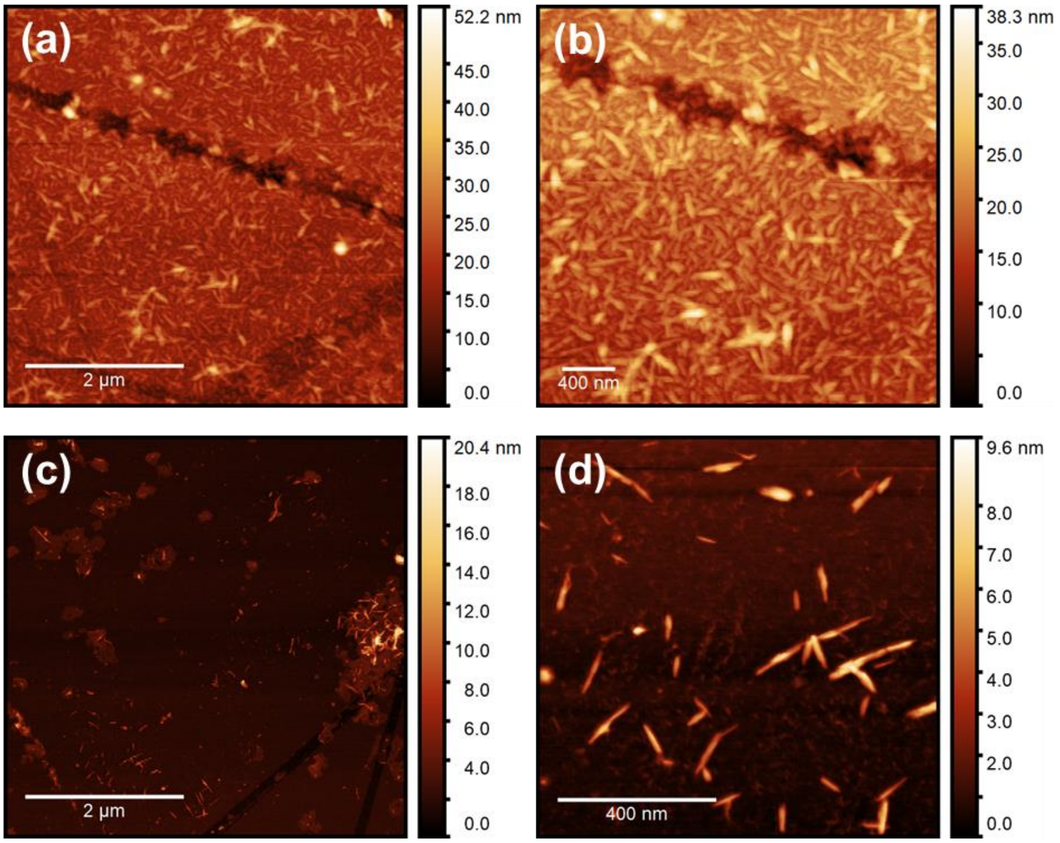

Figure S3. (**a**) Atomic force microscopy images of the nanocrystals. (**a**) and (**b**) CNCs with scanning areas of 5 × 5 μm² and 3 × 3 μm², respectively. (**c**) and (**d**) CCNCs with scanning areas of 5 × 5 μm² and 1 × 1 μm², respectively.

Table S2. The list of the power-law ($\eta = K\dot{\gamma}^{n\text{-}1}$) parameters obtained by fitting the curves in Figure 2a and 2d. The R^2 value of each linear fitting was also listed. The flow indexes (n) of both inks were close to zero. Some flow indexes were slightly less than zero. This result may come from the accuracy of measuring viscosity or the complex flow behavior of concentrated nanocrystals suspension.

**CNCs (wt%)**	**5**	**7**	**10**	**12**
**K (Pa·s^n)**	3.94	24.83	98.86	414.95
**n**	0.065	0.04	−0.026	-0.058
**R^2**	0.993	0.992	0.996	0.999
**CCNCs (wt%)**	**2.5**	**5**	**7**	
**K (Pa·s^n)**	6.44	107.40	289.73	
**n**	0.23	−0.065	0.025	
**R^2**	0.996	0.989	0.994	

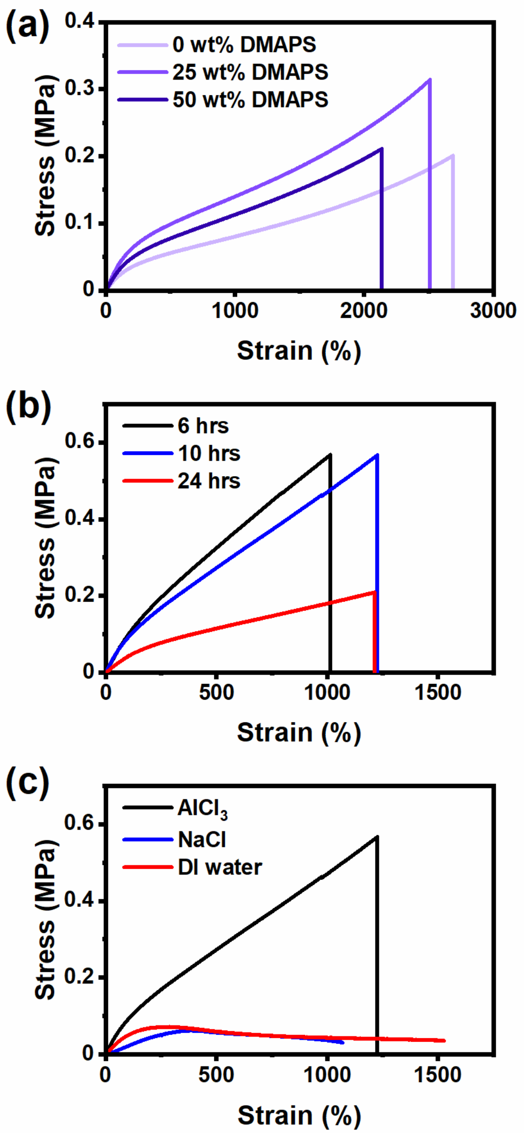

Figure S4. Mechanical properties of hydrogels using various recipes. (a) Different weight ratios of AA/DMAPS. Samples were tested before the addition of Al^{3+}. (b) Different immersing times in the aqueous solution of 0.5 M $AlCl_3$. (c) Hydrogels immersed in deionized water, the aqueous solution of 0.5 M NaCl, and the aqueous of 0.5 M $AlCl_3$ for 10 hours. All hydrogels were prepared by using 5 wt% of CCNCs.

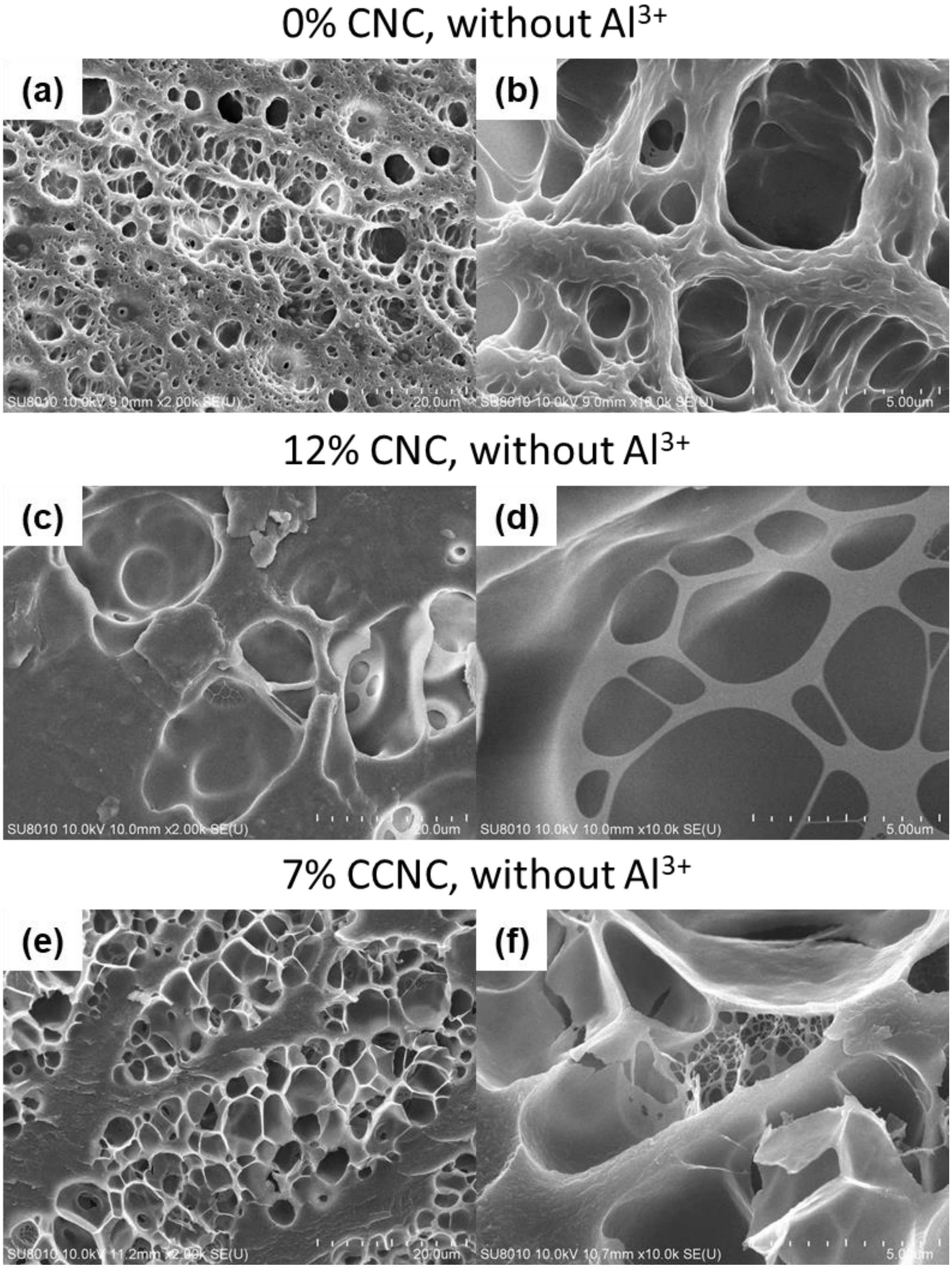

Figure S5. SEM images of the nanocomposite hydrogels. (**a**) and (**b**), hydrogels without any nanocrystals. (**c**) and (**d**), hydrogels with 12 wt.% of CNCs. (**e**) and (f), hydrogels with 7 wt.% of CCNCs. All samples were analyzed before immersing into the aqueous solution of $AlCl_3$.

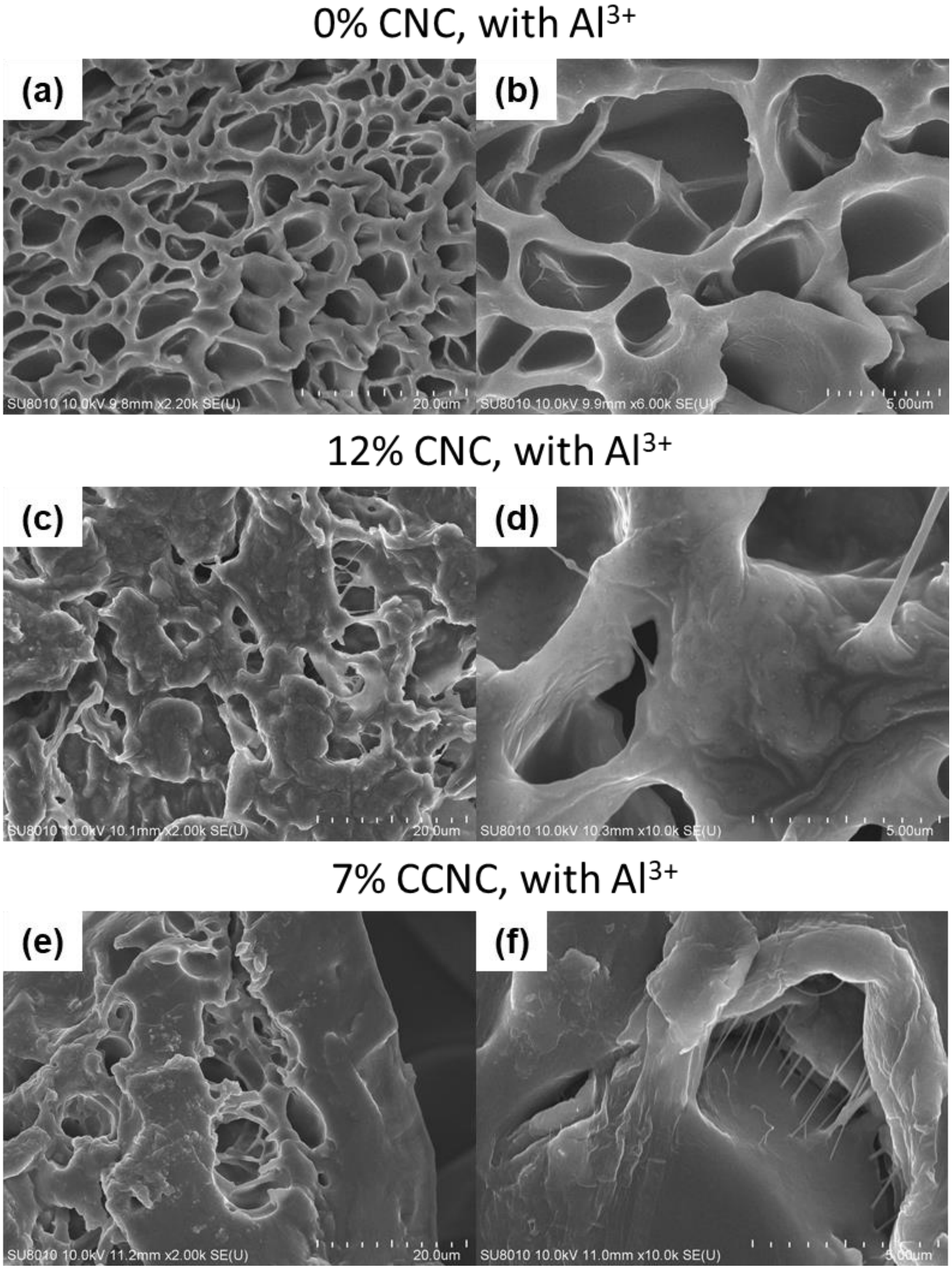

Figure S6. SEM images of the nanocomposite hydrogels. (**a**) and (**b**), hydrogels without any nanocrystals. (**c**) and (**d**), hydrogels with 12 wt.% of CNCs. (**e**) and (**f**), hydrogels with 7 wt.% of CCNCs. All samples were immersed in a 0.5 M aqueous solution of $AlCl_3$ for 10 hours.

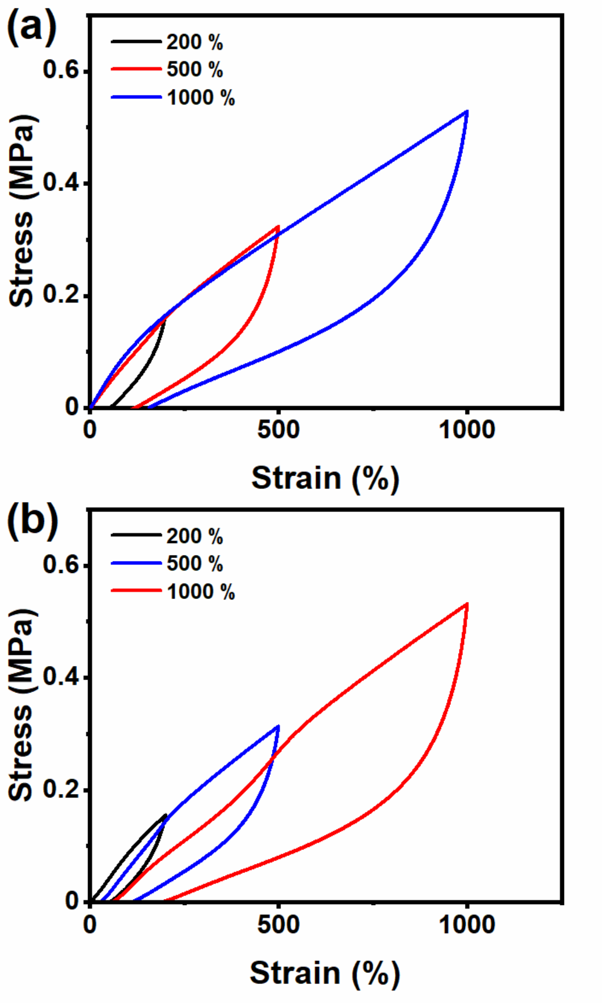

Figure S7. Loading-unloading tests and self-recovery of the nanocomposite hydrogels with 7 wt.% of CCNCs. (**a**) Cyclic tensile test of hydrogels with 7 wt.% of CCNCs to different strains. (**b**) Successive cyclic tensile tests of the same hydrogel with 7 wt.% of CCNCs to various strains. All samples were immersed in a 0.5 M aqueous solution of $AlCl_3$ for 10 hours.

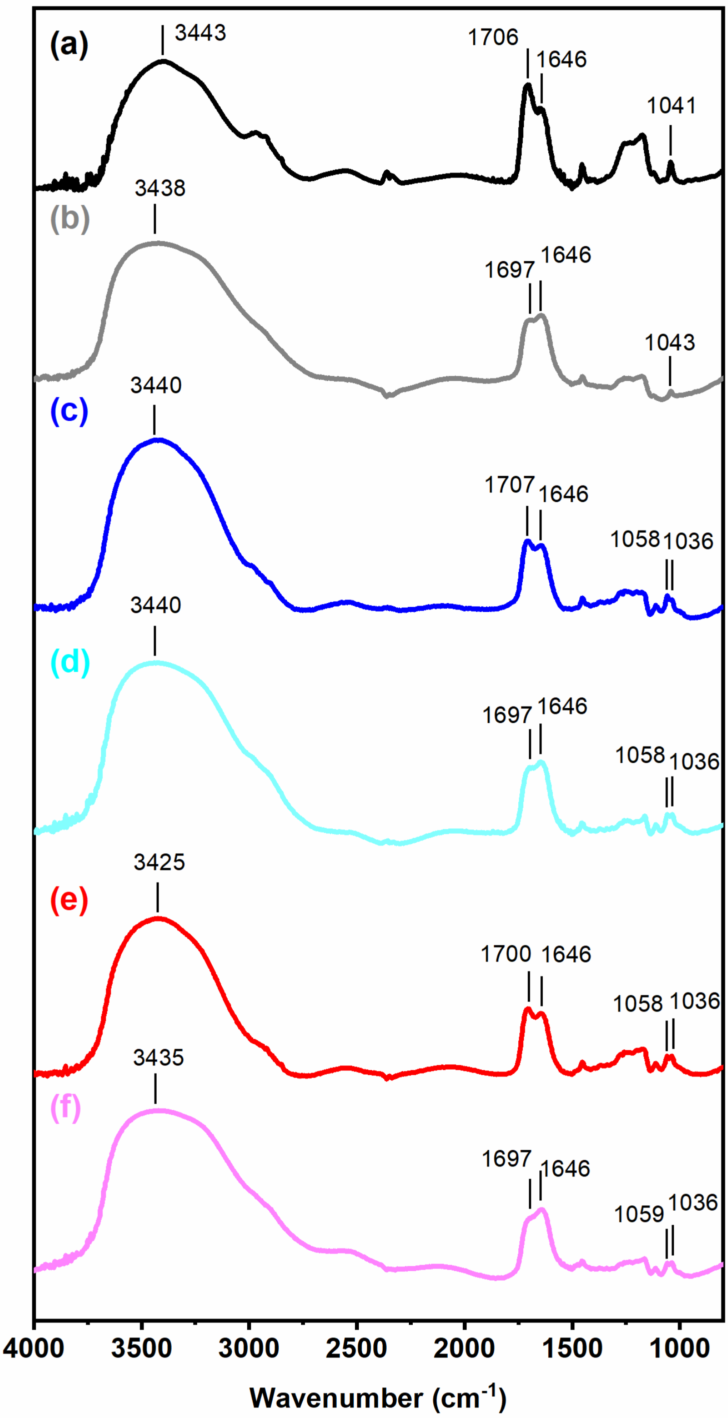

Figure S8. FT-IR spectra of different hydrogels. (**a**) Hydrogel without the addition of Al^{3+} and nanocrystals. (**b**) Hydrogel without nanocrystals after the addition of Al^{3+}. (**c**) Hydrogel with 12 wt.% of CNCs before the addition of Al^{3+}. (**d**) Hydrogels with 12 wt.% of CNCs after the addition of Al^{3+}. (**e**) Hydrogel with 7 wt% of CCNCs before the addition of Al^{3+}. (**f**) Hydrogels with 7 wt.% of CCNC after the addition of Al^{3+}.

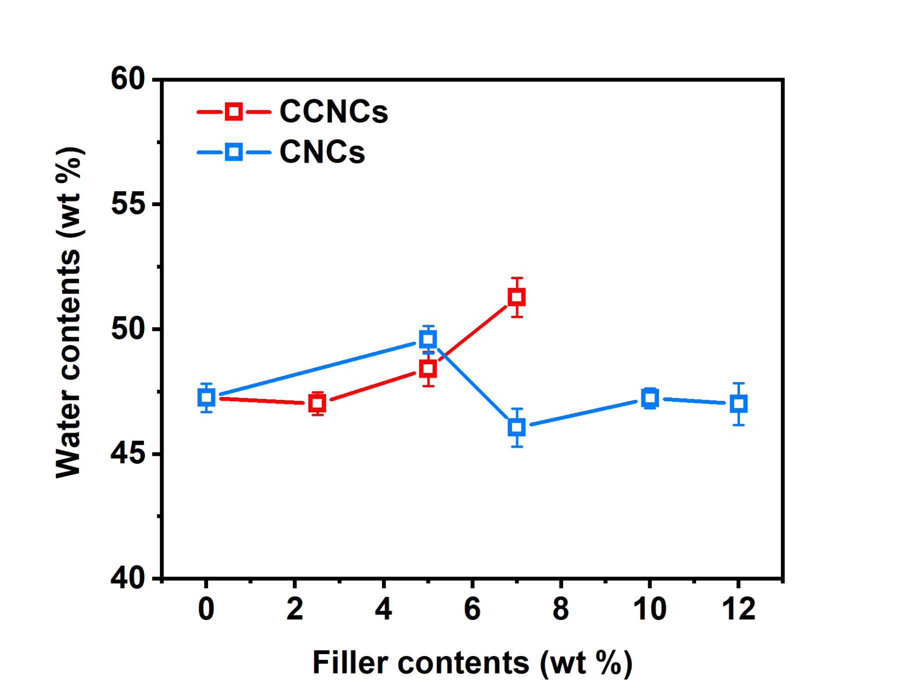

Figure S9. Water contents of the nanocomposite hydrogels with different concentrations of CNCs or CCNCs.

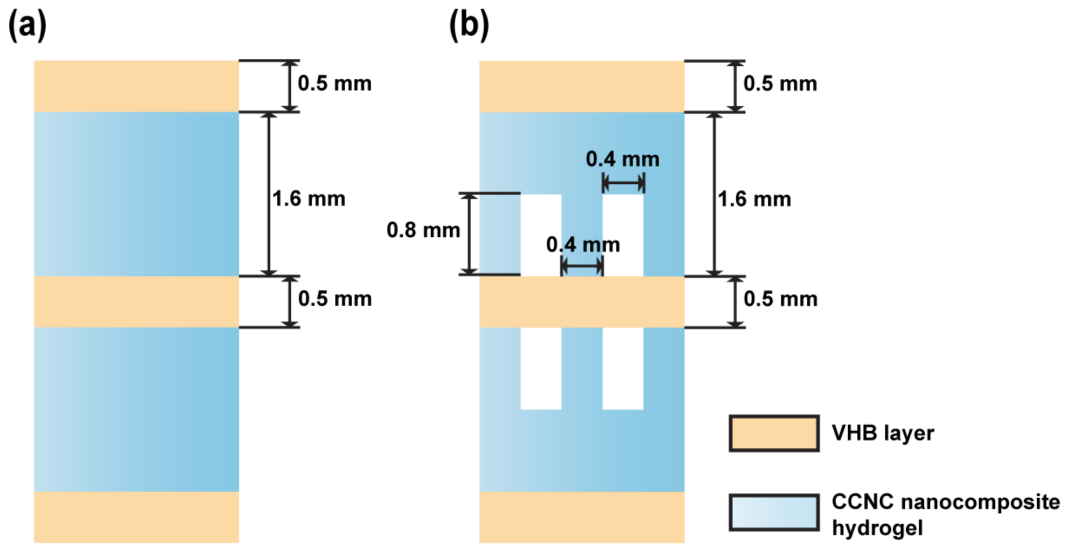

Figure S10. Design of the tactile sensors. (**a**) Thin film. (**b**) Grooved hydrogels. For the grooved structures, only two units are shown.

References

1. Li, M.-C.; Mei, C.; Xu, X.; Lee, S.; Wu, Q. Cationic surface modification of cellulose nanocrystals: Toward tailoring dispersion and interface in carboxymethyl cellulose films. *Polymer* **2016**, *107*, 200-210, doi:10.1016/j.polymer.2016.11.022.
2. Siqueira, G.; Kokkinis, D.; Libanori, R.; Hausmann, M.K.; Gladman, A.S.; Neels, A.; Tingaut, P.; Zimmermann, T.; Lewis, J.A.; Studart, A.R. Cellulose nanocrystal inks for 3d printing of textured cellular architectures. *Adv. Funct. Mater.* **2017**, *27*, 1604619, doi:10.1002/adfm.201604619.
